# From Morphology to Gene Expression Profiling in Mycosis Fungoides: Is It Still a Diagnostic Challenge?

**DOI:** 10.3390/diagnostics15091089

**Published:** 2025-04-25

**Authors:** Alessandra Filosa, Gerardo Cazzato, Elisa Bartoli, Elena Antaldi, Federica Giantomassi, Matteo Santoni, Gaia Goteri

**Affiliations:** 1 Biomedical Science a Public Health Department, Section of Pathological Anatomy, Polytechnic University of Marche Region, 60121 Ancona, Italy; 2 Department of Precision and Regenerative Medicine and Ionian Area, Bari University, 70121 Bari, Italy; 3 Pathological Anatomy Unit, Azienda Ospedaliero Universitaria delle Marche, 60126 Ancona, Italy; elisa.bartoli@ospedaliriuniti.marche.it (E.B.); elena.antaldi@ospedaliriuniti.marche.it (E.A.); 4 Oncology Unit, Macerata Hospital, Azienda Sanitaria Territoriale 3, 62100 Macerata, Italy

**Keywords:** mycosis fungoides, inflammatory dermatoses, histopathological features, molecular biology, artificial intelligence

## Abstract

**Background**: We herein review the most important clinico-pathological features of mycosis fungoides (MF). These evolving clinico-pathological aspects are paired with innovative therapeutic schemes. Moreover, we indicate cutaneous lymphomas as a new frontier of artificial intelligence application. **Methods**: We encompass new diagnostic and prognostic data derived from the recent medical literature describing the possible histological features which could be the targets of deep learning in conjunction with available clinical data. **Results**: In spite of decades of research, MF diagnosis still represents the most challenging debate from a dermatopathologist’s point of view. Genetic alterations have been identified mainly in late stages of the disease, and their importance for disease initiation is still unclear. The exploration of the genome-wide expression of individual genes in skin samples may be useful in elucidating MF pathogenesis and improving early diagnosis, while artificial intelligence could offer the possibility of searching for biomarkers of disease progression. **Conclusions**: MF still deserves the name of the ‘great imitator’, both clinically and histopathologically. The goal of summing up all the clinico-pathological information before reaching a final diagnosis is the approach needed to reach diagnostic accuracy, especially in early MF cases. It is advisable to think of the most common clinical presentations, to be aware of the most common histopathological features, and to interpret the results of ancillary studies only in the right clinico-pathological context.

## 1. Introduction

The fifth edition of the World Health Organization Classification of Haematolymphoid Tumours evaluates all hematopoietic neoplasms under a more complete perspective encompassing also genetic and molecular data, thus including in schematic classification systems old diagnostic categories and new ones [1]. What about mycosis fungoides (MF)? MF truly represents the oldest nosological entity in the field of cutaneous lymphomas, after the first description by the French dermatologist Alibert more than two centuries ago [1,2]. The fifth WHO classification has not effaced analytical MF pathogenesis, maintaining the original categorization of the disease among T-cell cutaneous lymphomas. Since the first case series reported in the medical literature, MF is still the most common type of cutaneous lymphoma, representing almost 50% of all primary cutaneous lymphomas [3]. The incidence of the disease showed a worldwide regular increase in recent years with about 6–7 new cases/1 million with regional variations. The disease is more common in adults and elderly patients, with a male to female ratio of 2:1. MF is definitely not rare among children and adolescents as well, and in this age group it represents the most common type of cutaneous lymphoma. There is evidence of a higher incidence in Black patients, with a younger average age of onset for Blacks than for Caucasians [4]. Although the epidemiological and nosological data are almost unchanged over the years, what about the contribution of gene expression profile data in MF pathogenesis and disease evolution? Is morphology still a reliable diagnostic approach? Is there an evolving contribution of the molecular techniques for MF prognosis? In spite of decades of research, MF pathogenesis remains unknown. A genetic predisposition may be suspected in some cases. A familial occurrence has been occasionally reported in MF with disease onset in identical twins [4]. A greater allele frequency of HLA DQBI*03 has been identified among MF patients who are Israeli Jewish [4,5]. Association with prolonged exposure to different allergens and chronic skin disorders had been originally tagged as possible promoting factors, but no other epidemiological study has ultimately renewed these hypotheses. MF cases have been rarely observed after solid organ transplantation, suggesting MF as an immune-suppression-driven lymphoproliferative disorder in such cases [4]. We herein review the most important clinico-pathological features of the disease, encompassing new diagnostic and prognostic data derived from the recent medical literature on MF. These evolving aspects of clinico-pathological characterization are paired with innovative therapeutic schemes. Moreover, we indicate cutaneous lymphomas as a new frontier of artificial intelligence application, describing the possible histological features which could be the targets of deep learning in conjunction with available clinical data.

## 2. Clinical Appearance

The clinical setting of MF patients is well established and the classical presentation of the disease encompasses the evolution of patches, plaques, and tumors [2]. Patches are generally seen admixed with, and sometimes topographically connected to, plaques and tumors in patients with advanced disease. MF patches are characterized by variably sized, slightly erythematous lesions in sun-protected areas, particularly the buttocks, and the breasts in women. Scaling is variable, in patches, as is itching. Plaques are more infiltrative lesions with a tough consistency and a more purple macroscopic appearance. MF tumors may be solitary, or multiple and diffuse, and are commonly observed in combination with typical patches and plaques. The MF clinical course is usually protracted over years [1,2,3]. More than 90% of patients with early MF do not progress to the tumor stage, particularly because therapeutic options became more effective over time. Patients with advanced MF who are in complete remission may relapse with patches, plaques, or tumors. Involvement of the mucosal areas may occasionally be observed in advanced stages. Erythroderma can develop in patients with every MF stage and should be distinguished from Sezary syndrome (SS), which is a distinct nosological entity in the WHO classification with different molecular features, prognosis, and thus different therapeutic schemes [3].

## 3. At Microscopic Glance

**Figure 1:**
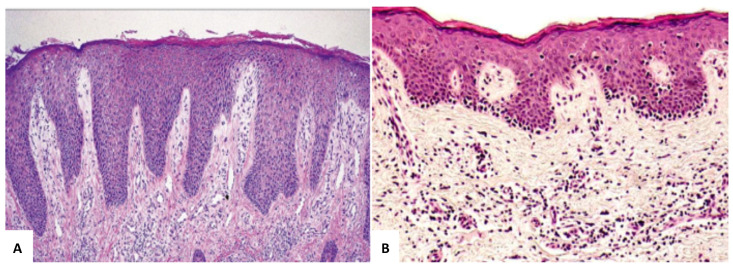
Epidermal hyperplasia with superficial perivascular infiltrate without epidermotropism in psoriasiform chronic dermatitis (**A**) (×40). Linear epidermotropism of lymphocytes among basal keratinocytes in early MF stage (**B**) (×40).

Histopathologically, MF is characterized by an infiltrate of atypical lymphocytes with convoluted irregular nuclei, commonly displaying a T-helper CD4+ phenotype. Early MF lesions usually reveal a patchy lichenoid or band-like infiltrate of small-sized atypical lymphocytes in a fibrotic papillary dermis [6,7,8]. Epidermotropism is considered the diagnostic hallmark of the disease: solitary small- to medium-sized atypical lymphocytes are usually found among basal keratinocytes at an early stage, with intraepidermal collections of so-called ‘cerebriform’ lymphocytes (Pautrier’s microabscesses or Darier’s nests) only in a minority of early MF cases [2]. The absence of intraepidermal lymphocyte abscesses in patch-stage MF could be a consequence of local skin-directed treatment modalities (e.g., local steroids, ultraviolet irradiation) that patients commonly receive before the initial skin biopsy. This ‘linear’ epidermotropism is, however, helpful in distinguishing a chronic superficial dermatitis (Figure 1A) from initial MF (Figure 1B). It is important not to forget that in more advanced lesions, the infiltrate becomes dermal-based, typically losing its epidermotropism as well. On the other hand, pagetoid epidermotropism is otherwise present as a diagnostic rule in the MF variant called Pagetoid Reticulosis and in MF cases with cytotoxic phenotype [6]. The dermatopathologists’ efforts in reaching diagnostic accuracy in early MF cases have culminated over years in a simple list of histopathological criteria [7,8]. The choice of specific criteria relies on the reproducibility of such features that include both epidermal and dermal modification, as in the following:

- Intraepidermal collections of lymphocytes.
- Basilar epidermotropism (lymphocytes aligned along the dermo-epidermal junction).
- Intraepidermal lymphocytes larger than lymphocytes in the dermis.
- Large numbers of epidermotropic lymphocytes in areas of scant spongiosis (so-called disproportionate epidermotropism).
- Intraepidermal lymphocytes with “haloed” nuclei (lymphocytes with slightly larger nuclei surrounded by a small halo).
- Band-like or patchy lichenoid infiltrates of lymphocytes.
- Expanded papillary dermis with slight fibrosis.

Less recent studies focusing on MF histology and immunohistochemical diagnostic aspects succeeded also in highlighting some secondary aspects commonly seen in association with the most typical ones. For example, some accessory cells are described in association with MF atypical lymphocytes: CD1a+ Langerhans cells are usually increased in the epidermis and dermis in early MF lesions as a possible consequence of a recruitment immune input from neoplastic cells. Sparse eosinophils may be present as immune by-standers, but in large numbers they are not a common finding in MF patches and plaques and their presence is otherwise particularly helpful in distinguishing early MF from subacute dermatitis [6,7,8]. Different histopathological patterns have been described in early MF, and many of them parallel those observed in inflammatory dermatoses, which represents the most challenging differential diagnosis [6]. Dermatopathologists should take into consideration also the unusual histopathological patterns less commonly observed in early MF phases: these include the presence of prominent spongiosis with interface damage, sometimes with several necrotic keratinocytes, similar to erythema multiforme; melanophages in the papillary dermis (typically seen in hyperpigmented MF); extravasation of erythrocytes evoking lichen aureus or lichenoid purpura; prominent epidermal acanthosis and papillomatosis as in lichen simplex chronicus; and significant papillary dermis sclerosis mimicking lichen sclerosus. In rare cases, subcorneal neutrophil abscesses may be visible and may correspond to a pustular clinical appearance. Edema is not typical of MF and, when present, is often used to favor an alternative diagnosis of dermatitis. Moreover, in some young patients, the histopathological features of evident clinical patches may resemble those observed in pityriasis lichenoides et varioliformis acuta [2,6].

**Figure 2:**
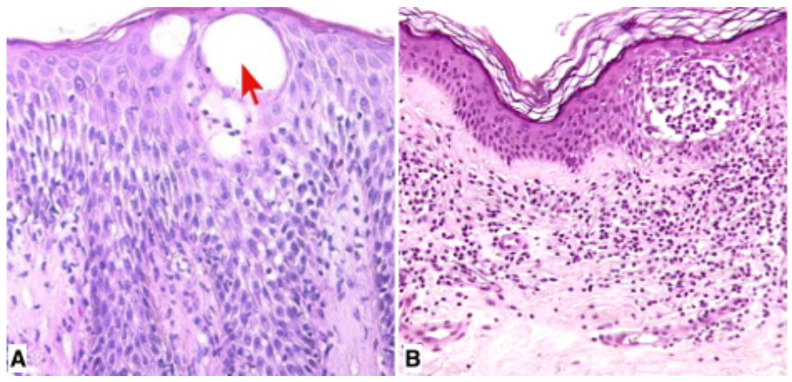
Intraepidermal spongiotic vesicle (red arrow) in subacute spongiotic dermatitis (**A**) (×60). Intraepidermal Pautrier’s microabscess in plaque-stage MF (**B**) (×40).

In most early MF cases, typical histological features are still not overt and a definitive diagnosis can be made only after a careful correlation with the clinical features of the disease. In such cases, the histopathological report should mention the possibility of MF diagnosis only with a mandatory clinico-pathological correlation. In doubtful cases, it is advisable to take multiple concurrent biopsies from morphologically different lesions and, if necessary, to repeat biopsies after a brief period of local treatment withdrawal or on recurrent lesions in order to check if the histopathological features are overlapping and stable [9]. MF plaques are histopathologically well-established lesions and are characterized by a dense, band-like infiltrate of atypical lymphocytes within the upper dermis. Epidermotropism shows both the same pattern described for MF patches and a predominance of small/medium pleomorphic lymphocytes, with intraepidermal microabscesses more commonly observed. Usually, it is not particularly challenging in the MF plaque stage to distinguish spongiotic vesicles (Figure 2A) from true Pautrier’s intraepidermal abscesses (Figure 2B). In MF tumors, a nodular and diffuse infiltrate is found within the superficial and deep dermis, sometimes involving the subcutaneous fat. Epidermotropism may be lost with an evident grenz zone in the papillary dermis. In some cases, flat MF tumors may present with a predominantly interstitial infiltrate of atypical lymphocytes [1,2]. Prominent involvement of the subcutaneous fat may be observed in some cases that simulate a subcutaneous panniculitis-like T-cell lymphoma. Angiocentricity and angiodestruction may be present, evoking the picture of natural killer/T-cell cytotoxic lymphomas from which they are morphologically truly indistinguishable. In tumor-stage patients treated with Brentuximab, xanthomatous infiltrates have been observed overwhelming the previous neoplastic infiltrates, but the real immunological pathogenic mechanism of this finding is still debatable [2]. In advanced stages, large pleomorphic and anaplastic lymphocytes and true immunoblasts may become the only types of cells within the neoplastic infiltrate. This situation has been detected in more than 50% of patients with tumor-stage MF and represents a large-cell transformation defined by the presence of large lymphocytes exceeding 25% of the infiltrate, or of large lymphocytes forming microscopic nodules. Clusters of large, atypical lymphocytes may sometimes be found also in plaques, and rarely in long-standing MF patches, usually in patients having concurrent tumors at other sites [2,8]. A high proliferative index and positivity for p53 is useful to confirm MF large-cell transformation. Transformed large cell may express CD30 or can be CD30-. Negativity for CD30 has been related to a poor prognosis in transformed MF. On the other hand, MF with CD30+ large-cell transformation should be distinguished from cutaneous anaplastic large-cell lymphoma and lymphomatoid papulosis (LyP) but, although morphologically challenging, such cases can be correctly interpreted in the clinical context [2].

The pathogenetic mechanisms underlying MF evolution and oncogenic transformation include not only intrinsic factors such as tumor gene mutation but also extrinsic events with environmental factors playing pivotal roles. Within all MF types of lesions, non-neoplastic cells such as macrophages, fibroblasts, dendritic cells, mast cells, and myeloid-derived suppressor cells definitely represent the tumor microenvironment (TME) with a complex interplay exerting a stepwise role in disease evolution. TME is variably composed of immune and nonimmune cells that can support tumor growth and survival and that, on the other hand, are responsible for the suppressed antitumoral immune response [2]. Besides conventional presentations, several variants of MF have been described. We here mention three variants which were included separately in the WHO European Organization of Research and Treatment of Cancer (WHO-EORTC) classification of cutaneous lymphomas and also in the 2017 revision of the WHO classification of tumors of hematopoietic and lymphoid tissues. We refer to folliculotropic (or pilotropic) MF, localized pagetoid reticulosis (Woringer–Kolopp), and granulomatous slack skin [1,2,3]. The purpose of this review includes offering a detailed description of these clinical-pathological variants.

## 4. Immunohistochemistry: The First Ancilla

Neoplastic lymphocytes in MF usually show a T-helper phenotype with βF1+, TCRγ−, TCRδ−, CD3+, CD4+, CD5+, CD8−, and TIA1-. In a subset of cases, neoplastic cells show a cytotoxic T phenotype with CD4−, CD8+, and TIA-1+ lymphocytes, or a γ/δ T-cell phenotype (βF1−, TCRγ+, TCRδ+, CD3+, CD4−, CD5+, CD8+, TIA-1+). These alternative immunohistochemical patterns do not demonstrate clinical and/or prognostic differences [2,10,11]. In such cases, correlation with the clinical features is mandatory, in order to exclude a diagnosis of aggressive cytotoxic lymphomas such as cutaneous aggressive epidermotropic CD8+ cytotoxic T-cell lymphoma or cutaneous γ/δ T-cell lymphoma [11]. Progressive loss of associated T-cell markers, such as CD2, CD5, and CD7, in CD4+ T-cells in repeated skin biopsies also supports the diagnosis of MF. Among them, the loss of CD2 and CD5 is rarely found in early MF. A decreased CD7 expression in less than 10% of neoplastic lymphocytes was reported to be 41–80% sensitive and 93–100% specific for MF diagnosis. Lack of such T-cell markers may be identified only in epidermal lymphocytes, as the number of tumor cells in the dermis is limited in most early MF. Not only morphological assessment but also interpretation of the immunohistochemical expression of T-cell markers is challenging in early MF. When examining immunohistochemical slides in possible early MF, CD4 to CD8 ratio evaluation should take into consideration the expression of CD4 by Langerhans cells and histiocytes. Early MF rarely displays an aberrant CD4+/CD8+ or CD4−/CD8− phenotype. These last cases may be positive for PD-1, a marker of follicular helper cells, with rare MF cases showing the complete phenotype of follicular helper T-cells (PD-1+, Bcl-6+, CXCL13+, CD10+) [2]. As an adjunctive observation, a follicular helper T-cell phenotype has been detected also in MF biopsies with large-cell transformation, while TIA-1, granzyme B, and perforin are positive only in cytotoxic MF or, sometimes, in tumor lesions with variable expression also of CD56, even in some biopsies with conventional CD4 positivity. These results represent a caveat because such cases should not be classified as cytotoxic lymphoma, but as tumor-stage MF with a cytotoxic phenotype [1,2,3]. In exceptional cases, an aberrant CD20 expression can be observed in neoplastic T lymphocytes. Moreover, humoral MF biopsies may show a prominent amount of reactive CD20+ B lymphocytes, with germinal center formation, masking the true T-cell nature of the neoplastic infiltrate. These cases should not be misinterpreted as B-cell lymphoma, although it should be remembered that exceptional cases of composite lymphoma have been observed. B-cell lymphomas composite with mycosis fungoides are mostly cases of B-cell chronic lymphocytic leukemia colonizing MF lesions [1,2,11]. In a diagnostic perspective, particularly for early MF cases, some minor immunohistochemical studies recently came to our attention. These studies try to overcome the difficulty in differential diagnosis between early MF and benign inflammatory dermatoses caused by the lack of tumor-cell-specific markers. Thymocyte selection-associated high mobility group box factor (TOX), belonging to the DNA-binding factors, regulates the positive selection of T-cells. TOX expression disappears from CD4-positive T-cells before they exit the thymus. Early studies reported TOX to be a tumor-cell-specific marker of CTCLs including early MF; TOX was expressed in tumor cells of CTCLs but has not been detected in inflammatory cells of benign inflammatory dermatoses. More recent studies support this hypothesis, with positive TOX expression identified in most (74%) MF cases and in a minority (32%) of benign dermatoses and normal skin. Therefore, TOX expression can be an adjunctive diagnostic marker for early MF, similar to the loss of pan-T-cell markers, and might be added in the pathological diagnostic algorithm. Moreover, cell adhesion molecule 1 (CADM1) was recently reported to be a potential diagnostic marker also in MF. Yuki et al. [12] revealed that 55 of 58 MF cases, including 34 early MF cases, showed CADM1 expression in more than 5% of neoplastic lymphocytes, while CADM1 expression was found in less than 5% of non-neoplastic lymphocytes in all 50 biopsies of inflammatory dermatoses. Fibrinogen-like protein 2 (FGL-2) is a serin protease that promotes mechanical cleavage of prothrombin into thrombin. FGL-2 is expressed on the surface of monocytes/activated macrophages and endothelial cells and is secreted by CD4 + CD8 + lymphocytes of the peripheral blood. After having been initially reported to be increased in various types of solid tumors, FGL-2 protein has been tested as a potential biomarker for early MF diagnosis by studying its activity in peripheral blood mononuclear cells of MF patients and comparing the results with those observed in patients with inflammatory dermatoses. The early MF subgroup patients showed 1.4-fold higher FGL-2 activity, making this blood test a potential marker for early MF [13].

## 5. Molecular Genetics and MF

Genetic alterations have been identified mainly in late stages of the disease, and their importance for disease initiation is still unclear. The TCR genes are clonally rearranged in the majority of MF cases, but the percentage of positive cases depends on the selected technical method of analysis. Polymerase chain reaction (PCR) analysis is more sensitive than Southern blot analysis and is more frequently used in the diagnosis of early MF [14,15,16,17]. Some recent reports showed that clonal TCR gene rearrangement was demonstrated in 83% of early MF cases by PCR analysis [2,14,15,16,17]. However, due to high sensitivity, the presence of monoclonality demonstrated by PCR can be seen in some inflammatory dermatoses, because oligoclonal accumulation of T-cells can occur also in these non-neoplastic lesions. Detection of identical clones from two different sites of skin biopsy was reported to be highly specific for MF [15,16,17]. On the other hand, TCR sequencing allows better characterization of the T-cell infiltrate and is superior to conventional polymerase chain reaction studies in the analysis of clonality. Recently, the application of next-generation high-throughput sequencing (NGS) to the detection of malignant clones in CTCL has been proposed as a more reliable method for analysis of T-cell clonality [18,19], offering true advances over standard PCR methods and providing also reproducible quantitative data on the number of neoplastic cells in a given infiltrate. Interestingly, comprehensive whole-exome sequencing showed that the majority of the MF samples presented multiple TCRγ, TCRα, and TCRβ ‘clono-types’, suggesting that the initial malignant transformation may occur before TCRβ or TCRα rearrangements at the level of T-lymphocyte progenitor rather than in mature memory T-cells. The third complementarity determining regions (CDR3) of TCRβ and TCRγ genes can be sequenced and the total amount and frequencies of the individual T-cell clones can be quantified with the unique nucleotide sequences of each clone’s CDR3 regions detected. The sensitivity of NGS is obviously superior to that of the PCR method, but expanded T-cell clones have rarely been detected in benign infiltrates. With 5% and 25% top clone frequency thresholds, the specificities for CTCL diagnosis were 95% and 100%, and sensitivity 89% and 50% [18]. Finally, microRNA (miR) profiles have been widely studied in CTCL, and since the first report in 2011, increased evidence of miR dysregulated expression has been reported also for early MF stages [20]. The exploration of the genome-wide expression of individual genes in skin samples may be useful in elucidating MF pathogenesis and improving the early diagnosis of MF patients [21,22].

## 6. Ongoing Consideration: What About Artificial Intelligence?

In most early MF cases, the diagnosis should be reported by a pathologist only as a possibility, sometimes suggesting a close follow-up and eventually a repeated biopsy. Thus, in such cases, an important role should nowadays be left to artificial intelligence, which must be available in pathology labs for some practical hints about MF patients [22,23]. Recently, some papers have underlined such a possibility in cutaneous lymphoma, focusing on models that could differentiate benign inflammatory dermatoses from initial MF patch stages. Some authors reached a 75.9% classification accuracy, which is the level of reproducibility we have between two different pathologists facing an early MF diagnosis [24]. Artificial intelligence could offer the possibility of rapidly identifiable biomarkers for disease progression, such as CD30 and GATA-3, and to obtain a quite exact quantification of these established biomarkers [25].

## 7. Conclusions

In spite of decades of research, MF diagnosis still represents the most challenging debate from a dermatopathologist’s point of view. This ancient disease nowadays reasonably deserves the name of the ‘great imitator’, both clinically and histopathologically, and the goal of summing up all the observations before reaching a final diagnosis is the only approach needed to reach a satisfying diagnostic accuracy in most early MF cases. It is advisable to think of common clinical presentations, to be aware of clues from histopathological features, and to interpret the results of ancillary studies only in the context of clinical and pathological findings. Discussion of doubtful cases with the clinician is always mandatory for dermatopathologists and progressive molecular research is desirable to predict disease evolution. All technological efforts should support a conclusive diagnosis in order to refine patient prognosis and to tailor therapy schemes to the best of our knowledge of the most widespread primitive cutaneous lymphoma.
